# Supplementary material for: Radiomics and Clinicopathological Characteristics for Predicting Lymph Node Metastasis in Testicular Cancer
Source: Cancers (Basel). 2023 Nov 29;15(23):5630. doi: 10.3390/cancers15235630 (PMC10705534; doi:10.3390/cancers15235630)
Supplement: Supplementary file 1 [file cancers-15-05630-s001.zip › cancers-2677934-supplementary.pdf]

SUPPLEMENTAL MATERIALS

Supplemental Table S1. Imaging and Preprocessing.

Acquisition and Reconstruction	
All patients underwent contrast-enhanced CT according to standard clinical scanning protocols:	
Tube voltage 100 kV - 120 kV with automatically calculated tube current	
Slice thickness ≤3 mm	
Field of view 300 - 350 mm	
512 × 512 matrix	
Brilliance 64 or Brilliance 16 CT scanners	
Radiomics analysis was performed on the portal-phase acquisition, delayed 60-70 s after starting contrast injection of weight-matched dose Ultravist® 370 (Bayer Schering Pharma.	
For image segmentation and analysis, all reconstructed images were retrieved from the hospital's picture archiving and communication system (PACS).	
**ROI-Segmentation, Texture Analysis and Feature Extraction**	
Software	Three-dimensional region of interest segmentation, texture analysis and feature extraction were performed using Mint Lesion™ software (version 3.8.4, mint Medical GmbH, Heidelberg, Germany).
**Settings of the Radiomics Feature Extraction**	
Bin Method	FBN
Bin Amount	32
LoG Filter	0
LoG Sigma	2
Matrix Aggregation Method	3D Average
Directions	
Resample Filter	1
Resample Spacing X	1
Resample Spacing Y	1
Resample Spacing Z	1
Second-Order Distance	1
Threshold Filter	0
**Image Biomarker Computation / Parameters**	
**Radiomics Features of First Order**	
**Intensity-Based Features**	
	Intensity Minimum
	Intensity Maximum
	Intensity Range
	Intensity Mean
	Intensity Variance
	Intensity Standart Deviation
	Intensity Skewness
	Intensity Kurtosis
	Intensity Energy
	Intensity P10th

Intensity P25th
Intensity P50th
Intensity P75th
Intensity P90th
Intensity Root Mean Square
Intensity Mean Absolute Deviation
Intensity Robust Mean Absolute Deviation
Intensity Median Absolute Deviation
Intensity Coefficient Variation
Intensity Quartile Coefficient Dispersion
Intensity Interquartile Range 44

Intensity Histogram Features

Histogram Minimum
Histogram Maximum
Histogram Range
Histogram Mean
Histogram Variance
Histogram Standart Deviation
Histogram Skewness
Histogram Kurtosis
Histogram Entropy
Histogram Uniformity
Histogram Mean Absolute Deviation
Histogram Robust Mean Absolute Deviation
Histogram Median Absolute Deviation
Histogram Coefficient Variation
Histogram Quartile Coefficient Dispersion
Histogram Interquartile Range
Histogram P10th
Histogram P25th
Histogram P50th
Histogram P75th
Histogram P90th
Histogram Minimum Histogram Gradient Intensity
Histogram MaximumHistogram Gradient Intensity

Radiomics Features of Second Order:

Gray Level Co-Occurrence Matrix (GLCM) Features

Joint Maximum
Joint Average
Standart Deviation
Joint Variance
Joint Entropy
Difference Average
Difference Variance
Difference Entropy
Sum of Averages
Sum of Variance
Sum of Entropy

Angular Second Moment
Contrast
Dissimilarity
Inverse Difference
Inverse Difference Normalised
Inverse Difference Moment
Inverse Difference Moment Normalised
Joint Maximum
Joint Average
Standart Deviation
Joint Variance
Joint Entropy
Difference Average
Difference Variance
Difference Entropy
Sum of Averages
Sum of Variance
Sum of Entropy
Angular Second Moment
Contrast
Dissimilarity
Inverse Variance
Correlation
Auto Correlation
Cluster Shade
Cluster Prominence
Cluster Tendency
Information Correlation 1
Information Correlation 2
Inverse Variance 41

Supplemental Table S2. Machine learning predictive models.

- We used four classic machine learning algorithms to identify the best radiomics model for predicting lymph node metastases in testicular cancer: Random Forest (RF), Light Gradient Boosting Machine (LGBM), Support Vector Machine Classifier (SVC), and K-nearest neighbors (KNN) classifiers. The open-source Python machine learning library Scikit-learn was used to implement the algorithms (1).
- The Random Forest algorithm (RF) (2) is an ensemble classifier that produces multiple decision trees using randomly selected subsamples of the data set. The prediction is achieved by averaging the predictions of all decision trees.
- The LGBM (Light Gradient Boosting Machine) (3,4) is a gradient boosting framework that uses a histogram-based learning approach. It's designed for speed and performance, using light memory and parallel computing to handle large datasets and provide accurate predictions.
- The Support Vector Machine classifier (SVC) is a powerful classification algorithm that works by finding the best hyperplane to separate different classes in the data. It aims to maximize the margin between classes, making it highly effective for both linear and non-linear classification tasks (5).

- Also known as "instance-based" learning, because the hypotheses are built from the training instances, the K-Nearest Neighbours (KNN) algorithm (6,7) is based on dis-tance calculations between instances. The basis of KNN is the calculation of distances between instances, which are the labels of the training instances. Classification is based on the labels of the nearest neighbours.
- To improve the model's performance and maximise the area under the receiver operating characteristic curve (AUC-ROC), the optimal hyperparameters of the model were determined using a grid search procedure (8).

**Hyperparameters determined by grid search**	
Random Forest	'max_depth': 8 and criterion 'gini'
K-nearest Neighbour	Neighbours: 7
Support Vector Machine classifier	'nu' = 0.5,
Light Gradient Boosting Machine	'boosting_type': 'gbdt', 'learning_rate': 0.1, 'min_child_samples': 20

**Software for Model Development**	
Python Random Forest	'The machine learning-based feature selection and construction of the clinical and radiomics model were conducted using custom-developed software implemented with the Python Scikit-learn package (Python version 3.10, Scikit-learn version 0.23.3, *http://scikit-learn.org/*) (1,8).

Supplemental Table S3. Clinicopathological Characteristics of the Patients.

**Average Age** (Range)	35.2 ± 9.4 Years (18–63)
**Histological Type**	
Seminoma	60 Patients (66 %)
Non-Seminoma	31 Patients (34%)
**Tumor Classification (T Status)**	
T1a	64 (70%)
T1b	27 (30%)
**Tumor Marker**	
AFP positive	21 Patients (19%)
B HCG positive	40 Patients (44%)
AFP und B HCG positive	10 Patients (11%)
BMI (Range)	25.9 ± 4.6 (19.3 –43.9)
**Patients' Status in 6-Year Follow up**	
Complete remission (CR)	81 (89 %)
Relapse of Disease (RD) with Metastatic Lymph Nodes	10 (11 %)

Seminoma	6 Patients
Non-Seminoma	4 Patients

Supplemental Table S4. Performance of the Radiomics-only, Clinical-only and Combined Clinical-Radiomics Models of all Classifiers.

Classifier	AUC (95% CI)	Accuracy	Precision	Recall	F1 Score
**Random Forest**					
Radiomics-only	0.92 ± 0.04	0.85 ± 0.05	0.85 ± 0.05	0.85 ± 0.11	0.85 ± 0.05
Clinical-only	0.88 ± 0.04	0.79 ± 0.07	0.89 ± 0.04	0.65 ± 0.09	0.75 ± 0.06
Combined clinical–radiomics	0.95 ± 0.03	0.87 ± 0.06	0.89 ± 0.04	0.86 ± 0.13	0.87 ± 0.06
**LGBM**					
Radiomics-only	0.93 ± 0.03	0.85 ± 0.04	0.86 ± 0.05	0.84 ± 0.09	0.85 ± 0.04
Clinical-only	0.86 ± 0.05	0.73 ± 0.05	0.79 ± 0.06	0.63 ± 0.07	0.69 ± 0.05
Combined clinical–radiomics	0.93 ± 0.05	0.83 ± 0.07	0.87 ± 0.05	0.80 ± 0.14	0.82 ± 0.08
**SVM**					
Radiomics-only	0.71 ± 0.05	0.68 ± 0.06	0.65 ± 0.06	0.80 ± 0.07	0.71 ± 0.05
Clinical-only	0.69 ± 0.12	0.63 ± 0.07	0.70 ± 0.15	0.50 ± 0.20	0.55 ± 0.11
Combined clinical–radiomics	0.71 ± 0.06	0.68 ± 0.07	0.65 ± 0.07	0.80 ± 0.07	0.71 ± 0.06
**KNN**					
Radiomics-only	0.51 ± 0.04	0.80 ± 0.03	0.17 ± 0.13	0.12 ± 0.08	0.14 ± 0.09
Clinical-only	0.48 ± 0.06	0.79 ± 0.06	0.05 ± 0.12	0.05 ± 0.12	0.05 ± 0.12
Combined clinical–radiomics	0.73 ± 0.04	0.67 ± 0.04	0.67 ± 0.05	0.69 ± 0.08	0.67 ± 0.04

References:

1. Pedregosa F, Varoquaux G, Gramfort A, Michel V, Thirion B, Grisel O, et al. Scikit-learn: Machine learning in Python. the Journal of machine Learning research. 2011;12:2825–30.

2. Breiman L. Random Forests. Machine Learning. 2001 Oct 1;45(1):5–32.

3. Alzamzami F, Hoda M, El Saddik A. Light gradient boosting machine for general sentiment classification on short texts: a comparative evaluation. IEEE access. 2020;8:101840–58.

4. Ke G, Meng Q, Finley T, Wang T, Chen W, Ma W, et al. Lightgbm: A highly efficient gradient boosting decision tree. Advances in neural information processing systems. 2017;30.

5. Cervantes J, Garcia-Lamont F, Rodríguez-Mazahua L, Lopez A. A comprehensive survey on support vector machine classification: Applications, challenges and trends. Neurocomputing. 2020;408:189–215.

6. Pisner DA, Schnyer DM. Support vector machine. In: Machine learning. Elsevier; 2020. p. 101–21.

7. Laaksonen J, Oja E. Classification with learning k-nearest neighbors. In IEEE; 1996. p. 1480–3.

8. Agrawal T, Agrawal T. Hyperparameter optimization using scikit-learn. Hyperparameter optimization in machine learning: make your machine learning and deep learning models more efficient. 2021;31–51.
